# Is there a protective effect of normal to high intellectual function on mental health in children with chronic illness?

**DOI:** 10.1186/1753-2000-4-3

**Published:** 2010-01-20

**Authors:** Hilde K Ryland, Astri J Lundervold, Irene Elgen, Mari Hysing

**Affiliations:** 1 Centre for Child and Adolescent Mental Health, Uni Health, University of Bergen, John Lunds plass 3, 5020 Bergen, Norway; 2 Department of Biological and Medical Psychology, University of Bergen, Jonas Lies vei 91, 5009 Bergen, Norway; 3 Department of Pediatrics, Haukeland University Hospital, 5021 Bergen, Norway

## Abstract

**Background:**

High intellectual function is considered as a protective factor for children's mental health. Few studies have investigated the effect of intellectual function on mental health in children with chronic illness (CI). The aim of the present study was twofold: First, we asked if *normal to high *intellectual function (IQ) has a protective effect on mental health in children with CI, and secondly, if this effect is more substantial than in their peers (NCI).

**Methods:**

The participants were selected among children who participated in the Bergen Child Study (BCS): 96 children with CI (the CI-group) and 96 children without CI (the NCI-group). The groups were matched on intellectual function as measured by the WISC-III by selecting the same number of children from three levels of the Full Scale IQ Score (FSIQ): "very low" (<70),"low" (70 to 84), or "normal to high" (>84). CI was reported by parents as part of a diagnostic interview (Kiddie-SADS-PL) that also generated the mental health measures used in the present study: the presence of a DSM-IV psychiatric diagnosis and the score on the Children's Global Assessment Scale.

**Results:**

The risk of a psychiatric diagnosis was significantly lower for children with a normal to high FSIQ-level than for children with a very low and low FSIQ-level in the CI-group as well as in the NCI-group. The group differences were statistically non-significant for all three FSIQ-levels, and the effect of the interaction between the group-variable (CI/NCI) and the FSIQ-level was non-significant on both measures of mental health.

**Conclusion:**

The present study showed a protective effect of normal to high intellectual function on children's mental health. This protective effect was not more substantial in children with CI than in children without CI.

## Background

Children with chronic illness (CI) have an increased risk of mental health problems [1]. This was confirmed in a population based study, the Bergen Child Study, showing that children with CI had a higher risk of emotional and behavioural problems and obtained a psychiatric diagnosis more frequently than children without CI [2]. Mental health in children with CI is affected by a range of factors, such as socioeconomic status (SES) [3], condition severity, functional status, the child's coping skills, as well as intellectual function [4]. The identification of risk and protective factors is important to improve treatment and preventive efforts.

Intellectual function (IQ) is a factor that is known to have a considerable effect on a child's mental health. First of all, it is well known that children with an IQ-level below 70 have an increased risk of mental health problems [5]. This increased risk is also shown in children with what is often referred to as a borderline intellectual disability [6-10]. On the other hand, high IQ is considered as a protective factor for children's mental health [5].

The association between IQ and mental health has also been studied in children with CI. This was demonstrated in a study by Howe and collaborators, showing that the higher risk of behavioural problems in children with neurological disorders compared to children with other chronic illnesses was partly mediated by decrements in IQ [11]. In a study by Goodman and Graham, children with hemiplegia with below average IQ (70-99) had a 57% rate of mental health problems, compared to 28% in children with above average IQ [12]. In children with sickle cell disease, the risk of behaviour problems has been shown to decrease with higher levels of intellectual functioning [13].

Living with a CI commonly implies that a child has to cope with a higher level of stress than his or her physically healthy peers, due to stressors originating from the physical condition and its consequences [14]. Accordingly, one should expect that a protective effect of normal to high IQ is even more substantial in children with CI than in their peers. This was shown in a study by Perrin and collaborators, including 96 healthy children and 91 children with different chronic conditions, aged 7 to 18 years. All children obtained a score above 80 on the Peabody Picture Vocabulary Test (PPVT), and mental health was assessed by the ASEBA screening questionnaires (CBCL, TRF and YSR) [15]. As far as we know, the work by Thompson et al. [13] and Perrin et al. [15] are the only studies focusing on the protective effect of IQ in children with CI.

This motivated the present study to further explore the protective effect of *normal to high *IQ (>85) in a case-control selected sample from a population based study of primary school children aged 7-11 years, including a subsample of children with CI. The present study improves on previous studies by including a measure of intellectual function from a standardized test (WISC-III) [16] and measures of mental health from a validated clinical interview generating DSM-IV diagnoses (Kiddie-SADS-PL) [17] and a general function score (the Children's Global Assessment Scale) [18]. The aim of the study was twofold: First, we asked if normal to high IQ had a protective effect in children with CI, and secondly, if this effect was more substantial than in their peers (NCI).

## Methods

### The Bergen Child Study

The Bergen Child Study (BCS) is an ongoing longitudinal population-based study of children born in the years 1993-1995 in the Bergen and Sund municipalities in Norway. The protocol and population of the BCS is described in detail elsewhere [19,20], and only a brief summary will be given here.

The BCS included all children in the two municipalities attending the 2^nd ^to 4^th ^primary school grades in October 2002, when the children were 7 to 9 years old. The total number of children attending these grades was 9430 in Bergen and 222 in Sund. The first wave had a three-stage design. In the *first screening stage*, a four-page questionnaire, including the Strengths and Difficulties Questionnaire (SDQ) [21,22] and a question about chronic illness or disability, was sent to all parents and teachers. Parents of 74% of the children gave their consent to participate. A child was defined as screen positive if: (1) the SDQ total difficulties score exceeded the 90^th ^percentile cutoff according to parents or teachers, (2) there was a severe impairment according to parents or teachers on the SDQ impact section, or (3) the score on one of the other scales included in the questionnaire exceeded the 98^th ^percentile cutoff. The families of children defined as screen positives in the first stage and a random sample of screen negative children were invited to participate in the *second stage *of the BCS, with a participation rate of 44%. In this *second stage*, the parents were interviewed with the Development and Well-Being Assessment (DAWBA) [23]. In the *third stage*, an extensive clinical examination of a case-control sample was performed (N = 329). The sample included 97 children who obtained a psychiatric diagnosis according to the DAWBA, 207 children without any DAWBA diagnosis, and 25 children invited directly from the first screening stage.

The study was approved by the Regional Committee for Medical and Health Research Ethics Western Norway, and by the Data Inspectorate.

### Instruments

The procedure of the third stage included a test of intellectual function (Wechsler's Intelligence Scale for Children, 3^rd ^ed., WISC-III) [16] and a psychiatric diagnostic interview (Schedule for Affective Disorders and Schizophrenia for School-Age Children: Present and Lifetime Version, Kiddie-SADS-PL) [17], including an evaluation of the child's general level of functioning (The Children's Global Assessment Scale, CGAS) [18].

*Wechsler's Intelligence Scale for Children, 3^rd ^ed *(WISC-III) is designed to assess intellectual function in children and adolescents aged 6-16. The scale contains 13 subtests which generate scores of Verbal IQ, Performance IQ, and Full Scale IQ, as well as four factor scores [16]. WISC-III is a widely used test of intellectual function, with strong criterion validity [24]. In the present study, the WISC-III was administered and scored by well-trained and experienced test-technicians employed at a Neuropsychological Outpatient Clinic. Intellectual level was defined by the Full Scale IQ-score (FSIQ) according to Swedish norms [25]. The FSIQ was categorized into a *very low level *(FSIQ below 70), a *low level *(FSIQ ranging from 70 to 84), and a *normal to high level *(FSIQ equal to or above 85). The last category included both children with a FSIQ-level within the normal range (i.e. 85 to 115) and 15 children with a higher IQ-score (3 in the CI-group and 12 in the NCI-group).

*The Schedule for Affective Disorders and Schizophrenia for School Aged Children (6-18 years): Present and Lifetime Version *(Kiddie-SADS-PL) is a semistructured interview designed to evaluate current and past episodes of psychopathology in children (6-18 years old) according to the criteria of DSM-IV [17]. Research has shown that this version of the Kiddie-SADS gives an appropriate schedule to assess current, past, and lifetime diagnostic status in children [26], and that it generates 32 reliable and valid DSM-III-R and DSM-IV Axis I child psychiatric diagnoses [17]. In the present study, clinical psychologists and MDs trained by an experienced child psychiatrist in using the instrument conducted the interview, first with the parent(s) and later on the same day with the child. Immediately after the assessment of both informants the interviewer scored the diagnoses as definite, probable, in remission, or not present according to the Kiddie-SADS-PL schedule. When in doubt, cases were discussed with the psychiatrist in charge of the training procedure. In the present study we defined a psychiatric disorder as *any definite diagnosis*. As part of the Kiddie-SADS-PL-interview the parents were asked if the child had any physical illnesses or disabilities for which (s)he received or should receive regular care (for example asthma, epilepsy, diabetes).

*The Children's Global Assessment Scale *(CGAS) is a 100-point rating scale measuring the child's general level of functioning. Scores above 70 indicate normal function. The CGAS is considered a valid and reliable tool for rating a child's general level of functioning on a health-illness continuum, and is recommended as a supplement to syndrome-specific scales [18]. The CGAS is part of the Kiddie-SADS-PL interview.

### Participants in the present study

**Figure 1:**
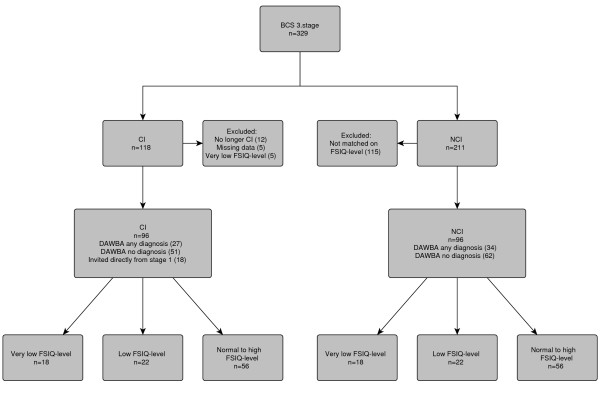
**Flow chart visualizing the selection procedure**. BCS = Bergen Child Study; CI = Chronic Illness; NCI = No Chronic Illness; FSIQ-level = Full Scale IQ-level; DAWBA = Development and Well-Being Assessment.

CI was defined as reported by parents on the questionnaire in the first stage of the BCS and confirmed by parents in the clinical interview in the third stage (i.e. still present), and only physical conditions were included. An experienced paediatrician categorized the reported illnesses (Table 1). Children who no longer met the criteria of a CI in the third stage (n = 12) were excluded from the present study. Another five children were excluded because of missing data (WISC-III and/or Kiddie-SADS). The FSIQ-levels of the remaining 96 children in the CI-group were used to select a matched group of children without CI (the NCI-group). Using the select random sample command in SPSS, an equal number of children with a very low, low, and a normal to high FSIQ-level were generated in both groups (Figure 1). The percentage of screen positive and screen negative children from the first stage of the BCS was 64.6% and 35.4%, respectively, in the CI-group, and 63.5% and 36.5% in the NCI-group.

**Table 1:** Reported chronic illnesses and disabilities (n = 96)

Neurological (n = 22)*	Atopic (n = 50)*	Somatic (n = 24)*
Epilepsy (8)	Allergies (41)	Skeletal disorders (11)
Migraine (6)	Allergy not specified (17)	Deformations of the foot (3)
Learning disabilities (6)	Pollen (15)	Cheilognathopalatochisis (2)
Cerebral palsy (4)	Animals (8)	Malformations (2)
Hydrocephalus (2)	Food (7)	Hypermobility of the joints (1)
Down syndrome (1)	Dust mite (4)	Disease in the hip (1)
William syndrome (1)	House dust (5)	Perthes disease (1)
Frontal lobe damage (1)	Nickel (1)	Scoliosis (1)
Sequela meningitis (1)	Vaccines (1)	Gastro-intestinal disorders
Brain tumour (1)	Asthma (37)	Reflux (4)
	Eczema (8)	Coeliac disease (3)
		Disease in the gallbladder (1)
		Disease in the liver (1)
		Sensory impairments (3)
		Visual deficit (2)
		Hearing deficit (1)
		Endocrine disorders (2)
		Hypothyreosis (2)
		Kidney disorder (1)
		Haemophiliac (1)
		Juvenile Rheumatoid Arthritis (1)
		Other (1)
		Malaria (1)

*Numbers add up to more than 22, 50 and 24 because some children had multiple diagnoses.

### Statistical analyses

For statistical analyses we used the SPSS version 15.0. Descriptive statistics were used to explore characteristics of the sample concerning gender, age, FSIQ and psychiatric disorders. Chi square tests were used to detect statistically significant differences in psychiatric disorders according to group (CI/NCI) and FSIQ-level.

Binary logistic regression analyses were conducted to further explore the impact of normal to high intellectual function on mental health, using group (CI/NCI) and FSIQ-level (very low, low, and normal to high) as predictors and any Kiddie-SADS diagnosis as the dependent variable. The analyses were conducted in the following way: First, the main effect of each predictor was explored (Model A). As the FSIQ-level variable had three categories and our focus was on the impact of a normal to high FSIQ-level, separate analyses were conducted with the very low and the low FSIQ-level as the reference group. Secondly, the interaction was explored (Model B). Main effects are presented as odds ratios (OR) with 95 percent confidence intervals and the interaction effect as chi square (*x*^2^).

A two-way between-groups analysis of variance (ANOVA) was performed to explore simultaneously the impact of group (CI/NCI) and FSIQ-level on the general level of functioning, as measured by the CGAS. Furthermore, one-way ANOVA was performed for the CI- and NCI-group separately to explore within-group differences between the three FSIQ-levels on the CGAS. Finally, an independent samples t-test was performed for the three FSIQ-levels separately to explore differences between the CI- and NCI-group on the CGAS.

## Results

### Characteristics of the sample

Boys constituted 64.6% of the sample in both groups, with an even distribution of age across the CI- (M = 9.7 years, *SD *= .95) and the NCI-group (M = 9.5 years, *SD *= .99). In the CI-group, mean FSIQ was 56.83 (*SD *= 12.28, range = 37-69), 79.00 (*SD *= 3.83, range = 72-84), and 97.73 (*SD *= 9.21, range = 85-128) within the three FSIQ-levels. The corresponding numbers in the NCI-group were 56.50 (*SD *= 9.02, range = 41-69), 77.95 (*SD *= 4.92, range = 71-84), and 101.91 (*SD *= 11.60, range = 85-126).

### Psychiatric disorders according to group (CI/NCI) and FSIQ-level

In this case-control selected sample matched on FSIQ-level, the overall percentage of psychiatric disorders was 51% in the CI-group and 35.4% in the NCI-group. This difference was statistically significant (*x*^2^(1) = 4.16, *p *= .04). Within the CI-group, the percentage of psychiatric disorders for children with a normal to high FSIQ-level (37.5%) was significantly lower than the percentage for children with a very low (72.2%) (*x*^2^(1) = 5.29, *p *= .02) and low FSIQ-level (68.2%) (*x*^2^(1) = 4.81, *p *= .03), while the difference between children with very low and low FSIQ-levels was non-significant. In the NCI-group, the percentage of psychiatric disorders for children with a normal to high FSIQ-level (23.2%) was significantly lower than the percentage for children with a very low (55.6%) (*x*^2^(1) = 5.23, *p *= .02) and low FSIQ-level (50.0%) (*x*^2^(1) = 4.14, *p *= .04), while the difference between the very low and low FSIQ-level was non-significant. The differences in percentages of psychiatric disorders between the CI- and the NCI-group were non-significant within each of the three FSIQ-levels (Table 2).

**Table 2:** Number of children with and without any psychiatric diagnosis (Kiddie-SADS-PL) according to FSIQ-level (n = 192)

CI-group	Very low FSIQ-level (<70)	Low FSIQ-level (70-84)	Normal to high FSIQ-level (>85)
Any psychiatric diagnosis, n (%)	13 (72.2)	15 (68.2)	21 (37.5)*
No psychiatric diagnosis, n (%)	5 (27.8)	7 (31.8)	35 (62.5)
Total, n (%)	18 (100)	22 (100)	56 (100)
**NCI-group**			
Any psychiatric diagnosis (%)	10 (55.6)	11 (50.0)	13 (23.2)*
No psychiatric diagnosis (%)	8 (44.4)	11 (50.0)	43 (76.8)
Total (%)	18 (100)	22 (100)	56 (100)

*The frequency of psychiatric disorders in children with a normal to high FSIQ-level was significantly lower than in children with a very low and low FSIQ-level.

Kiddie-SADS-PL = The Schedule for Affective Disorders and Schizophrenia for School Aged Children (6-18 years): Present and Lifetime Version; FSIQ-level = Full Scale IQ-level; CI-group = Children with chronic illness; NCI-group = Children without chronic illness.

The logistic regression analyses showed that children with CI had a twofold increased risk of psychiatric disorder compared to children without CI (OR = 2.04, 95% CI: 1.11-3.77). Children with a normal to high FSIQ-level had a significantly lower risk of psychiatric disorder compared to children with a very low (OR = .236, 95% CI: .11-.53) and low FSIQ-level (OR = .291, 95% CI: .14-.61) (Table 3). There was no significant interaction between CI and FSIQ-level with regard to risk of psychiatric disorder (*x*^2^(2) = .01, *p *= .99).

**Table 3:** Risk of psychiatric disorder (any Kiddie-SADS-PL diagnosis) by group (CI/NCI) and FSIQ-level (n = 192)

Predictor		OR	95% CI	p-value
Chronic illness		2.04	(1.11-3.77)	.02
Normal to high FSIQ-level versus very low FSIQ-level		.236	(.11-.53)	.0005
Normal to high FSIQ-level versus low FSIQ-level		.291	(.14-.61)	.001

Kiddie-SADS-PL = The Schedule for Affective Disorders and Schizophrenia for School Aged Children (6-18 years): Present and Lifetime Version; FSIQ-level = Full Scale IQ-level; CI = Chronic Illness; NCI = No Chronic Illness.

### General level of functioning according to group (CI/NCI) and FSIQ-level

The results of the two-way ANOVA showed a significant main effect for FSIQ-level regarding the general level of functioning (*F*(2, 186) = 30.96, *p *= .001) (partial eta squared = .25). Post hoc comparisons using the Tukey HSD test indicated that the mean CGAS-scores for children with a very low (*M *= 54.28, *SD *= 14.16), low (*M *= 68.95, *SD *= 15.90), and normal to high FSIQ-level (*M *= 77.34, *SD *= 15.75) were significantly different. The main effect for CI and the interaction effect were not significant.

Within the CI-group, the one-way ANOVA showed a significant difference at the *p *= .05 level on the CGAS-score for the three FSIQ-levels (*F*(2, 93) = 9.61, *p *= .001) (eta squared = .17). The post hoc test indicated that the mean score for children with a very low FSIQ-level (*M *= 54.83, *SD *= 15.63) was significantly different from the mean score of children with a low (*M *= 69.73, *SD *= 17.06) and normal to high FSIQ-level (*M *= 74.25, *SD *= 16.29). Children with a low and normal to high FSIQ-level did not differ significantly from each other. Within the NCI-group, the one-way ANOVA showed a significant difference at the p = .05 level on the CGAS-score for the three FSIQ-levels (*F*(2, 93) = 24.56, *p *= .001) (eta squared = .35). The post hoc test indicated that the mean scores for children with a very low (*M *= 53.72, *SD *= 12.97), low (*M *= 68.18, *SD *= 15.01), and normal to high FSIQ-level (*M *= 80.43, *SD *= 14.69) were significantly different.

**Figure 2:**
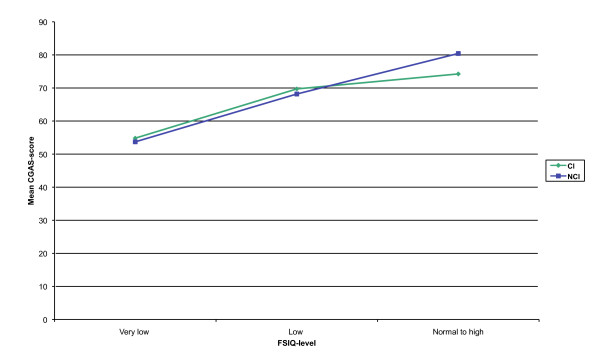
**Line graph showing mean CGAS-score for group (CI/NCI) and FSIQ-level (n = 192)**. CGAS = Children's Global Assessment Scale; CI = Chronic Illness; NCI = No Chronic Illness; FSIQ-level = Full Scale IQ-level.

Among children with a normal to high FSIQ-level, results of the t-test showed a significant difference on the mean CGAS-score between the CI-group (*M *= 74.25, *SD *= 16.29) and the NCI-group (*M *= 80.43, *SD *= 14.69; *t*(110) = 2.11, *p *= .04, eta squared = .004). Among children with a very low and low FSIQ-level, the t-tests showed no significant differences on the mean CGAS-score between the two groups (Figure 2).

## Discussion

In the present case-control study of primary school children, having a CI was associated with a higher risk of psychiatric disorder as assessed by the Kiddie-SADS-PL. The percentage of psychiatric disorders decreased and the general level of functioning increased as a function of higher FSIQ-level both in the CI-and the NCI-group. The protective effect of a normal to high FSIQ-level was not more substantial in children with CI, supporting an overall protective effect of normal to high intellectual function on children's mental health.

More than half of the children in the CI-group met the criteria of a psychiatric disorder, compared to a third of the children in the NCI-group. Thus, even when the two groups were matched on FSIQ-level, the overall percentage of psychiatric disorders was still significantly higher in children with CI. The estimated risk of psychiatric disorder in this case-control sample of children with CI is in accordance with the twofold increased risk of mental health problems in children with CI shown in a study of the whole population of the BCS [2]. Although the risk is similar, the overall percentage of psychiatric disorders is higher, as expected due to the selection of participants to this stage of the BCS.

The present study showed that children with a FSIQ-level between 70 and 84 had a similar risk estimate of psychiatric disorders as children with a FSIQ-level below 70 - a risk that was significantly higher than for children with a FSIQ-level of 85 or above. This finding is in accordance with the results of Goodman and collaborators, showing that healthy children with low IQ within the normal range (defined as WISC-R FSIQ in the range 70-89) had more behavioural problems compared to those with higher IQ-scores [10]. It is also consistent with the findings of Dekker and collaborators, showing that children with borderline intellectual disability (IQ-range 60-80) and those with moderate intellectual disability (IQ-range 30-60) had a similar rate and estimated risk of mental health problems that was significantly higher than for children with a higher level of intellectual function [7].

A protective effect of normal to high intellectual function was found both in the CI- and the NCI-group. Such an overall effect was contrary to what we expected from the stressors associated with CI and from the findings of Perrin et al. [15]. The differences between the results in Perrin and collaborators' and the present study may partly be ascribed to methodological factors. First of all, Perrin et al. had the focus on children with an IQ-score above 80, as it was measured by an unstandardized test of intellectual function (the PPVT). Secondly, the measures of mental health, the recruitment procedures and characteristics of the samples are quite different in the two studies. In Perrin et al.'s study, the healthy children were recruited from public and private schools, while the children with CI were recruited through generalist and specialist pediatric offices. The children participating in the present study were part of the same case-control sample selected from the BCS-population, with the same percentage of screen positive and screen negative children in the CI- and the NCI-group. Furthermore, the two groups in our study were matched on FSIQ-level. Consequently, the CI- and the NCI-group in the present study were probably more similar on critical variables than the corresponding groups in Perrin and collaborators' study.

### Strengths and limitations

The main strength of the study was the use of a standardized test of intellectual function (WISC-III) and a validated clinical interview generating DSM-IV diagnoses (Kiddie-SADS-PL). Moreover, the study sample was drawn from a population of children from the second largest city of Norway and included both screen positive and screen negative children. An additional strength was the use of a comparison group matched on FSIQ-level.

Some limitations should be mentioned. First of all, the use of categorical IQ measures reduced the statistical power of our analyses. The categorical levels were included due to our focus on children with an IQ-level within the normal range and higher, and it should be mentioned that an analysis of the full range of FSIQ-scores did not change the results concerning the impact of IQ on mental health problems. Secondly, the IQ-distribution of the CI-group was skewed. Only 3 children had an IQ-level above the normal range (>115), compared to 12 children in the NCI-group. This skewness probably reflects what the case is for children with CI as a group: compared to their peers, they have a higher frequency of general and specific learning disabilities, which in turn is associated with lower mean IQ [5]. Finally, although the protective effect of normal to high IQ was not more substantial in children with CI in the present study, it is still an important protective factor in relation to risk of mental health problems in this group of children. However, IQ only explained some of the association between CI and mental health. In future studies we will include other factors considered important for the mental health of children with CI.

### Clinical implications

The present study showed that children with a normal to high FSIQ-level had better mental health than children with a very low and low FSIQ-level. The frequencies of psychiatric disorders were somewhat higher in the CI-group compared to the NCI-group within all three FSIQ-levels. Paediatricians and others working with children with CI should be aware of this increased risk of mental health problems and the need of psychological support not only for children with low IQs, but also for children with an IQ-score within the normal range of intellectual function.

## Conclusion

The present study showed a protective effect of normal to high intellectual function on children's mental health. This protective effect was not more substantial in children with CI than in children without CI. Future studies should validate the clinical significance of the present findings and include other potential protective factors in children with CI.

## Abbreviations

BCS: Bergen Child Study; CGAS: Children's Global Assessment Scale; CI: Chronic Illness; CI-group: Children with chronic illness; FSIQ: Full Scale IQ; IQ: Intellectual function; Kiddie-SADS-PL: Schedule for Affective Disorders and Schizophrenia for School Aged Children (6-18 years): Present and Lifetime Version; NCI-group: Children without chronic illness; WISC-III: Wechsler Intelligence Scale for Children, 3^rd ^Ed.

## Competing interests

The authors declare that they have no competing interests.

## Authors' contributions

HKR has been responsible for the data analysis and the writing of the manuscript. AJL designed and coordinated the study, supervised the data analysis and the writing process. MH has been responsible for creating data files, has supervised the data analyses and commented on the written drafts of the manuscript. IE was responsible for defining and categorizing the chronic illnesses reported in the study, and commented on written drafts of the manuscript. All authors have read and approved the final manuscript.
